# Religion, Spirituality, and Health Revisited: Bringing Mainline Western Protestant Perspectives Back into the Discourse—Theology’s “Seat at the Table”

**DOI:** 10.1007/s10943-023-01888-3

**Published:** 2023-08-16

**Authors:** András Béres

**Affiliations:** Kaposi Mór Teaching Hospital, Tallián Gy. 20-32, 7400 Kaposvár, Somogy County, Hungary

**Keywords:** Religion, Spirituality, Health, Theology, Christianity

## Abstract

Theological perspectives have been given short shrift in the literature on religion and health research. This study demonstrates how including different schools of mainline Western Protestant theological thought (evolutionist, correlationist, and dialectical) in the scientific process could contribute to clarifying controversies. The issue is not just theoretical: Theology can even challenge assumptions on elicitability and reproducibility. Theology perceives spirituality as a dialogue with the Total Other, thus making each encounter with the transcendent (not just the individuality of the person) unique and unpredictable. By accepting setbacks on a journey with wide-ranging aspirations, theology redefines health as the momentum of constant striving toward the divine spirit. Since these theological insights relate to interventions that affect patients’ intimacy, attempting to recognize the (albeit implicit) spiritual–theological standpoint of the patient and the self—and how these relate to authentic traditions of spirituality—appears to be an essential prerequisite for ethical spiritual intervention.

## Introduction

**Fig. 1:**
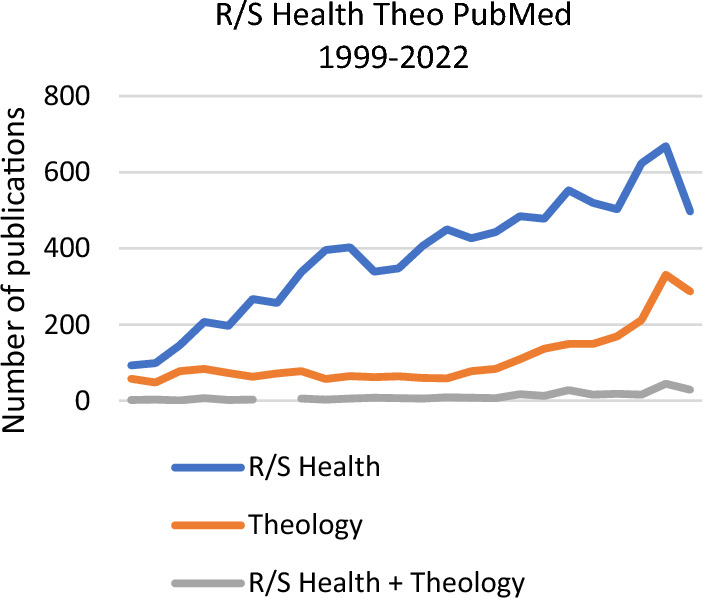
The number of publications in the field of religion (R), spirituality (S), and health from 1999 to 2022 is shown by the blue line; those mentioning theology are shown by the gray line. The number of publications in the field of theology is marked by the orange line. *Source:* PubMed database, February 2023 (Colour figure online).

In his exhaustive review encompassing more than 2300 relevant studies on the religion/spirituality (R/S)–health relationship from 1970 to 2010, Koenig (2012) presented a potentially beneficial effect of R/S on mental and physical health, including cardiovascular, cerebrovascular, neuroendocrine, and immunological aspects. His projection of the explosion in the number of further related studies also turned out to be well founded (Fig. 1). Thus, in line with the authors of the field, one could reasonably conclude that R/S is a major factor influencing health and that all possible efforts should be made to integrate R/S into clinical practice.

However, reading between the lines of this all-encompassing meta-analysis, the number of inconclusive studies and controversial outcomes is not to be underestimated, especially in terms of the scarcer studies that examine the impact of religion on physical health. Moreover, the exact method for implementing the findings poses several practical difficulties (Groopman, 2004) and remains a polemical issue to this day.

Interestingly, although traditionally delegated to the study of spiritual life for centuries—to the extent of once being designated as *mater studiorum*, the mother of the sciences (McGrath, 1994)—it seems the incorporation of the field of theology into the discourse on the effects of R/S on health has only recently followed the expansion of relevant literature (as seen in Fig. 1). Thus, it appears not only that Koenig’s prediction in his epochal meta-analysis turns out to be correct but also that Levin’s warning written in the exact same year remains valid: “[A] putative relationship between religious involvement and personal or population health is being treated especially by sociomedical and biomedical researchers as simply another garden-variety topic for sophisticated analysis. Religion is just more grist for the mill of structural-equation models, survival analyses, logistic regressions, and the like” (Levin & Meador, 2012, pp. 4–5). Levin continued, “So much effort is focused on the narrow details of respective studies and the application of cutting-edge methodologies and hardly on the sort of metaphysical questions (e.g., ‘What does this really mean?’) that occupied early investigators …. [I]n the understandable haste to get about one’s business, in a research sense, something important has been lost” (Levin & Meador, 2012, pp. 5–6). “[W]e have ignored our responsibility to connect our data to substantive, comprehensible, and recognizable features of normative religious belief and practice as experienced and sanctioned within the living faith traditions of our study subjects” (p. 9). The current article aims to contribute to filling this gap by presenting how religion, spirituality, and health relate to each other as seen through the lens of three major theological schools from the mainline of Western Protestantism. At times, this analysis also incorporates genuine Jewish insight and the progressive Catholic currents, both of which richly nourished the Western Protestant tradition. Thereby, this article proposes the theological perspective as a supplementary tool to interpret the often conflicting results in this field and addresses the abovementioned controversies.

### Scientific Studies on the R/S–Health Relationship: Basic Assumptions, Findings, and Open Questions

Most scientific studies on the R/S–health relationship operate with an implicit set of assumptions. Their overall results can be summarized as follows.

### Basic Assumptions

Most of the experimental studies on the effects of R/S on health implicitly assume that the phenomenon of R/S:

- Can be **measured** either retrospectively as a personality trait or prospectively as a quantifiable intervention, and
- Can be **elicited** in the context of an intervention.

The rationale behind this conception is that religious behavior, whether external or internal, produces at least some similar patterns across individuals, which are, therefore, quantifiable as parameters. For instance, attendance at religious events or self-reported religiosity seems to fit well in this conception: In principle, both are prone to precise measurements by means of direct observation or standardized questionnaires. Likewise, using classical spiritual texts, symbols, chants, or rites seems to render spiritual interventions within reach and reproducible upon demand at the will of the researchers.

### Results

Studies on the effects of R/S on health have produced conflicting results, as demonstrated by the following points:

- The balance is clearly on the *positive* side with respect to health benefits. Most studies report beneficial effects on a wide range of health outcomes (Koenig, 2012), including several measurable dimensions of mental health (on the one hand, increased well-being, happiness, hope, self-esteem, and a sense of control; on the other hand, a decrease in anxiety, depression, psychosis, suicide, social problems, and marital instability), health behaviors (e.g., more exercise, a better diet, less smoking, less risky sexual activity), and physical health (e.g., a positive effect on cardiovascular and cerebrovascular disease, Alzheimer’s, Parkinson’s (Otaiku, 2022), dementia, autoimmune disorders, endocrine disorders, cancer, pain, and overall mortality).
- Depending on the variable measured, a considerable share of these studies also *shows some negative effects*. For instance, some studies have found a negative impact of religion on mental health; 7% of the most rigorously performed studies revealed a positive relationship with depression, 10% reported greater levels of anxiety, 23% found a positive relationship with psychotic symptoms, and 9% indicated a positive relationship with neuroticism (Koenig, 2012). In particular, studies exploring the effects of religion on physical health point to a more nuanced picture with the possibility of harmful outcomes; 7% of studies found a positive link with coronary heart disease; 13% revealed higher levels of blood pressure; 11% reported more carotid artery thickening, placing individuals at greater risk of stroke; 21% reported negative ties with cognitive function; 7% showed mixed findings on immune response; 18% found worse outcomes, 15% reported mixed results for physical functioning; 8% found significant negative relationships between R/S and self-reported health; 20% indicated worse pain; and 5% demonstrated overall shorter longevity in groups with more R/S.

Including the theological viewpoint in the discourse helps the natural sciences by challenging basic assumptions while also explaining the conflicting nature of current findings.

### Theology: It Might Be Imperceptible, Yet it Remains Unavoidable

In reality, the spiritual nature of the phenomenon that researchers in the field of R/S deal with compels them to take a theoretical position regarding spirituality; hence, they are already engaged in theology, albeit their momentum could well go unnoticed. Indeed, not practicing theology when investigating the realm of the spiritual would be a paradox, like pretending that not communicating is possible, unaware of the fact that not sending clear communicative signals is also a form of communication. For instance, researchers who consider spirituality to be an elicitable, measurable, and replicable human phenomenon inadvertently take the stance of liberal theologians.

This theological movement—which remained active in the nineteenth century, along with the flourishing of the natural sciences in the post-Enlightenment period—took from Rationalism its confidence in human reason and its belief in the immanence of God. From Romanticism, the theological movement adopted the emphasis on feelings and the idea that self-consciousness, in a deep religious sense, becomes God-consciousness. Thus, the theological movement shifted along with Modernism in the direction of examining the Christian doctrine as a human psychological experience. In this mode of thinking, the religious experience becomes part of the human psyche, which can be studied like any other psychological factor (Schleiermacher, 1999).

Liberal theology remains an important school of thought to this day; however, it only represents a fraction of all theological thinking. This study proposes reincorporating the entire field of theology into mainstream discourse and presents three major currents that have shaped theology in recent decades and that could be especially relevant in terms of the R/S–health relationship.

### A Theological Analysis of Findings on the R/S–Health Relationship: Major Findings Re-Examined From the Angle of Three Different Western Protestant Theological Schools

The schools of thought presented below do not contradict—but rather complement—each other’s perceptions; they only differ in where they place an emphasis on the interpretations of their sources. Hence, they stress different aspects in the way they conceive of human health.

### From the Evolutionist School: Health as Growth into the Spirit—the Holy Spirit

German Lutheran theologian Wolfhart Pannenberg was a persistent advocate of theology as a rigorous academic discipline that is capable of fruitful exchanges not just with the social sciences but also with the natural sciences. He was also a committed supporter of evolutionist theology. Pannenberg (2006) stated, “At the end of the nineteenth century and in the first half of the 20th, sadly, Christian churches and theologians could not recognize that the teaching of evolution offers an unprecedented possibility to theology in regard to the possibility of its relationship with modern science. The fight against Darwinism was one of the mistakes resulting in the most serious consequences throughout the history of theology’s relationship with the sciences” (p. 39).

The roots of evolutionist theology can be traced back to the birth of *narrative theology*, a school of thought that highlights the linear, story-telling aspect of the Bible, specifically how, from one generation to another, God’s personality was gradually revealed to humankind. Indeed, textual research and archaeology indicate that the Bible (and especially the Old Testament, as used in its current form) is not just the outcome of additional writings regularly superimposed on previous texts. Instead, the entire text underwent regular, extensive editing by waves of scribes, especially in the first millennium BC. Thus, there seem to be at least four (sometimes contradictory) sources of text that reveal the totality of the Old Testament in the sense that the order of the sources was carefully selected and edited after the original sources were removed. Major historical milestones and sometimes traumatizing events in Jewish history made such revisions necessary, as new experiences brought about the need to reinterpret the Bible and to derive new insights into the way that יהוה was understood (translated as “JHWH,” these four letters refer to the secret pronunciation of the name of God; this phrase is so respected by Jews that it is not even permitted to be said aloud when the Torah is read and is therefore replaced by the word *Adonai*, i.e., “Lord,” when the Torah is read publicly) (Bright, 2000). Recently, a more practical attempt to reconstruct a genuine human narrative appeared in the epic project of Salopek’s (2013) “Out of Eden Walk.” Salopek is collecting personal narratives from ordinary people on a 21,000-mile (33,780 km) journey on foot, retracing the pathways of our ancestors, the first humans who migrated out of Africa and journeyed around the globe.

The evolutionist school of theology goes even further by proposing the idea that not just the Bible but history itself can be considered a form of revelation (Pannenberg, 2009a). The evolutionist school sees history as centered around the apparition of God in Jesus of Nazareth, giving an out-of-history (i.e., eschatological) orientation for the evolution of humankind toward Jesus as the Resurrected Christ. In other words, evolutionary theology considers the primary goal of history to be the evolution of humankind toward knowledge of God, not just in the sense of intellectual understanding but also of participation in the life of the divine. According to French Jesuit theologian-paleontologist Teilhard de Chardin (2008), “as early as in St. Paul and St. John we read that to create, to fulfill and to purify the world is, for God, to unify it by uniting it organically with himself” (pp. 293–294). God is “from this point of vantage in the heart of [the] matter, assuming the control and leadership of what we now call evolution” (p. 294). He stated that “through human socialization, whose specific effect is to involute upon itself the whole bundle of reflexive scales and fibers of the earth, it is the very axis of the cosmic vortex of interiorization which is pursuing its course” (p. 306).

As seen above, theology also adopted the term *evolution* from the natural sciences to use it equally as a notion of the organism’s progress toward a defined goal. The difference is that while the natural sciences define evolution’s goal as the survival of the species, theology elaborates on this concept of “survival” and uses it in the sense of knowledge of life in the widest, most religious sense of the word; that is, participation in the life of God’s spirit. According to Pannenberg (2006), if “[like Teilhard de Chardin,] we can consider life’s evolution as the process of the creation of life forms of increasing complexity and at the same time becoming increasingly introspective, then we can also state that in the succession of different forms of life by creatures, is expressed the increase in the shareholding of the divine spirit, of life’s spirit” (p. 99).

That said, Pannenberg (2009b) insists on a contrast that shaped the course of world history: The appearance of Jesus in the temporariness of world history can be considered a portent, an anticipation of the future, the intrusion of timelessness into the finite, while the notion of time remains linked with the sinful present in which living creatures eventually pass away (Polkinghorne, 2002). In contrast to the first finite appearance of Jesus of Nazareth in world history—the goal of which was to show humanity wallowing in the consequences of original sin and the *possibility* of a fuller life—the Christian faith traditionally used the term *parousia* (from the Greek παρουσία, literally meaning *presence*) to express the expectation of the second coming of Jesus as the Resurrected Christ, after which his presence in a re-created world is expected to last forever.

Thus, evolutionist theology views human life as a process of evolution, forming between the friction of two force fields—the timely and the eternal—shaping the human condition, and as a consequence, naturally bearing marks of pain and suffering while evolving toward its ultimate goal (Whitehead, 1978). This is the frame of thought in which evolutionist theologians interpret the relationship between R/S and health.

From evolutionist theologians’ point of view, the effect of religion on health is measurable, reproducible, and positive because, ultimately, *God’s intention* with humankind is to attract humans to Himself and to endow them with a full life in health. In the more nuanced exposition of this worldview, evolutionist theologians perceive human life, and accordingly human health, as being shaped by two contemporaneous and conflicting forces: the gradual degradation of the body as an outcome of original sin, and signs of health and joy while we are in psychological and spiritual harmony. In this sense, healing in this world is temporary; that is, even biblical healings are only anecdotical and of signal value because they hint at a precursory picture of a harmonious God–human relationship. This picture includes all its psychic and physical aspects, which will only be complete in the presence of the Resurrected Christ in the re-created world and resurrected bodies.

### From the Correlational School: Health as the Pursuit of Integrity

German-American Lutheran theologian Paul Tillich, who defined himself as a “boundary man”—an existential philosopher on the verge of the old and new, with heritage imbued with a sense of the sacred and the secular orientation of the new age (Unhjem, 2022)—introduced the phrase *method of correlation.* This term indicates a continuous effort to create links between insights from Christian revelation with the issues raised by existential, psychological, and philosophical analyses (Bowker, 2000). “In using the method of correlation, systematic theology proceeds in the following way: It analyzes the human situation out of which existential questions arise, and it demonstrates that the symbols used in the Christian message are the answers to these questions” (Tillich, 1951, p. 62). Or put more briefly, “[t]heology formulates the answers implied in divine self-manifestation under the guidance of the questions implied in human existence” (Tillich, 1951, p. 61).

The concept of participation in the life of the divine, as indicated in evolutionist theology, also appears in this school of thought, not as less than the very essence, but as the elementary driving force of faith: “Faith is not an opinion but a state. It is the state of being grasped by the power of being, which transcends everything that is, and in which everything that is, participates” (Tillich, 1980, p. 173).

However, the correlational school also emphasizes that this state of “being grasped by the divine” cannot simply be viewed as a matter of human choice and wittingly elicited, nor can it be taken for granted like an automatism. It also implies a process of learning in which questions and answers, as well as faith and doubt, alternate to form a constantly developing conversation between the limited and the sacred, the human, and the transcendent: “Being religious means passionately asking [questions] about the meaning of our existence and being willing to receive answers, even if the answers hurt” (Tillich, 1999, p. 1). More precisely, as Tillich (1981) writes, “the healing of the spirit is not possible by good will, because the good will is just that which needs healing. In order to be healed the spirit must be grasped by something which transcends it, which is not strange to it, but within which is the fulfillment of its potentialities. It is called ‘Spirit’ (with a capital *S*). Spirit is … the ground of our being and meaning” and “[t]his is the intention of religion, but it is not identical with religion. For as a function of the human spirit and as a realm of human activities, religion also stands under the dialectics of all life and under its ambiguities” (p. 57). In line with the above, “[r]eligious health is the state of being grasped by the Spirit, namely the divine presence, enabling us to transcend our religion and to return to it in the same experience,” while “[u]nhealthy religion is the state of being enslaved—socially or personally—by a concrete religious system, producing bigotry, fanaticism, inordinate self-destructive ecstasy, dogmatism, ritualism” (p. 57). As it appears from the lines above, the correlationist school emphasizes that the notion of health targets wider horizons than just the physiological state of body functions, and that the contribution of religion to health is dependent on the extent to which it facilitates connection to an authentic spiritual experience that transcends religious boundaries. This is the frame of thought in which correlationist theologians interpret the relationship between R/S and health.

The correlationist school underscores that religion and spirituality cannot be viewed as mere stimulants of better health because in terms of striving toward life in its completeness, the Christian message implies—and searches for—ways to courageously face life and death alike. The “courage to be” cannot be conceived of without the “courage to die.” That is to say, the courage to be involves accepting “oneself in spite of being unacceptable” (Tillich, 1980, p. 164) and “is rooted in a God who appears when God has disappeared in the anxiety of doubt” (p. 190). In this context “doubt is not the opposite of faith; it is one element of faith” (Tillich, 1957, p. 116). Ultimately, the courage to die entails testing “the courage to be. A self-affirmation which omits taking the affirmation of one’s death into itself tries to escape the test of courage, the facing of non-being in the most radical way” (Tillich, 1980, p. 169). Accordingly, the same applies for health and disease: “The concept of health cannot be defined without its relation to its opposite—disease. … In reality, health is not health without the essential possibility and the existential reality of disease. In this sense, health is disease conquered, as eternally the positive is positive by conquering the negative. This is the deepest theological significance of medicine” (Tillich, 1981, p. 60).

As Hungarian spiritual poet Weöres (2012) summarized (of note, the Hungarian word for “health” is *egészség*, which literally means “entirety”):

*Destroy your suppositions – belief shall be yourself*

*Break through your obstacles – the world shall be yourself*

*Compare your life and death – entirety shall be yourself*

The correlationists point out that religious life should not be seen as a constantly peaceful state of mind but as genuine striving, a quest for meaning, with potentially stressful experiences being part of the learning process. Meanwhile, at times, the message itself offers comfort in order to help us bear the burdens of disease and is not intended to heal us.

On the one hand, the complex and potentially stressful psychological aspects of spiritual interventions become tangible as soon as we take a closer look at the most basic phenomenon of an unexpected encounter with God. The earliest biblical narratives mention that the first spontaneous feeling that often arises during a meeting with the transcendent is deep existential fear. In the Old Testament, JHWH says to Moses, *“‘I will make all my goodness pass before thee, and I will proclaim the name of the* *Lord before thee; and will be gracious to whom I will be gracious, and will show mercy on whom I will show mercy.’ And he said, ‘Thou canst not see my face: for there shall no man see me, and live’”* (Exodus 33: 19–20, KJV). In the New Testament, when Peter realizes Jesus’s power following a miraculous fishing experience that results in an unmeasurable mass of fish, he falls down at Jesus’s knees and says, *“Depart from me, for I am a sinful man, O Lord!”* (Luke 5: 8, NKJV). It appears that looking God in the face, in contrast, brings into light one’s sinfulness and unworthiness, which is a potentially stressful experience. The psychological literature coined the term *mysterium tremendum et fascinans* to describe this sense of fascination mixed with elementary tones of fear, the first feeling to emerge, unconsciously, when the transcendent appears in ordinary life (Otto, 1923).

On the other hand, genuine Christian thinking includes an appreciation for acts of self-sacrifice (i.e., a deliberate decision to consciously act in contrast to a person’s health) up to the extreme of choosing death instead of life if a historical situation requires it. This can be the choice that Christian teaching recommends: *“Most assuredly, I say to you, unless a grain of wheat falls into the ground and dies, it remains alone; but if it dies, it produces much grain”* (John 12:24, NKJV). *“Greater love has no one than this, than to lay down one’s life for his friends”* (John 15:13, NKJV). In such circumstances, the spiritual message is not intended to restore our health but rather to help us bear the burden of loss.

From the above, it follows that from correlationist theologians’ perspective, the elicitability of spiritual interventions will depend on the *efforts* made to make interventions as personalized as possible, and the effect of R/S on health will depend on the degree to which the spiritual message is understood in terms of participants’ efforts to internalize it.

With psychological models of personality, the above quest can be conceptualized as a struggle between deeply rooted, acquired maladaptive schemes (as described in Young et al.’s (2003) model of schema therapy) and ancient, potentially sanative archetypical contents (as preempted by Jung’s (1959) model of the collective subconscious). Modern fMRI findings show that the same brain areas are activated during intense prayer and during communication with another person (Schjoedt et al., 2009), and we can see clearly that early maladaptive schemes acquired in relationships with parents or significant others (especially in early childhood) will have a potentially significant influence on a person’s spiritual life. Hence, we can anticipate that injuries acquired in the parent–child relationship will be projected onto the relationship with God, especially if one sees God as a parent.

In practice, it became the responsibility of the Church to facilitate the adaptation of the authentic Christian message in order to always make it intelligible and accessible for people of each respective time period. Whether the technical terms found in the theological literature and commonly used to mediate this message (e.g., “original sin,” Lat. *Peccatum originale*; “salvation,” Lat. *Salus*; “redemption,” Lat. *Resurrection*)—but which are hardly interpretable for a layperson (at least not in the sense intended when a term is originally introduced)—are to be kept and interpreted, or perhaps replaced by words that are easier to understand, remains a strongly contested topic to this day. While at first glance the latter seems more obvious, proponents of the former approach argue that many other skills used in everyday life also require basic preliminary studies, based on which it does not seem excessive for representatives of religious life to expect that people will learn a limited set of religious terms that have proven uniquely useful over the centuries.

### From the Dialectical School: Health as Harmony with the Other

If the early twentieth century was marked by the rise to prominence and gradual domination of liberal theology (Schleiermacher, 1999), with its tendency to depict the experience of God and religion as the outcome of a human effort, the dialectical school can be understood as a radical reaction to this view by accentuating the total otherness of the transcendent as compared with the human (hence the term *dialectical*). Swiss Reformed theologian Karl Barth posited that there is a radical, qualitative difference between God and humans that creates a gap between them, and only God is able to overcome it. From this, we derive an emphasis on the holy nature of Scripture, which is viewed as a revelation from the Total Other (Barth, 1933). The term “Word” refers to the Bible or to God Himself, because of which the dialectical school’s concept is also called the “Theology of the Word of God.”

“One of the absolutely crucial metaphors that Barth turned to again and again is the image of a tangent, i.e., a line touching a circle at only one point. This image, Barth thought, captured the theological reality of God’s relationship to the world. The first detail worth noticing in this picture is that God is totally other, not in any way part of, or knowable apart from, his choice to make himself known. Second, all of humanity’s efforts to strive toward God are doomed to fail since they are situated within a horizon that does not in any way include God. Third, God made and makes himself known at one point and at one point only—in God/man, Jesus” (Michaud et al., 2005).

Since God cannot be reached, reaching faith is also an impossible endeavor from a human perspective. Barth wrote, “Faith is not an art. Faith is not an achievement. Faith is not a good work of which some may boast while others can excuse themselves with a shrug of the shoulders for not being capable of it. It is a decisive insight of faith itself that all of us are incapable of faith in ourselves, whether we think of its preparation, beginning, continuation, or completion. In this respect, believers understand unbelievers, skeptics, and atheists better than they understand themselves. Unlike unbelievers, they regard the impossibility of faith as necessary, not accidental ….” (Barth, 1986, p. 38).

In this total showdown of any human attempt to understand and control God to any extent, Barth went so far as to reject religion as an obstacle, not a facilitator of authentic spirituality. As scholars would later summarize, “[Barth’s] theology depended on a distinction between the Word (i.e., God’s self-revelation as concretely manifested in Christ) and religion. Religion, according to Barth, is [a] human attempt to grasp at God and is opposed to revelation, in which God has come to humans through Christ. ‘Religion is the enemy of faith.’ ‘Religion is [a] human’s attempt to enter into communion with God on his own terms’” (Michaud et al., 2005). Furthermore, as Cobb (1962, p. 43) captures this characteristic feature of Barthian thinking, “God’s presence in Scripture is always [an] *event,* wholly uncontrollable and unpredictable from man’s side. Hence, the Roman Catholic understanding of the church’s authoritative definition of doctrine through its interpretation of Scripture and tradition must be vehemently rejected as denying God’s free sovereignty. In a similar way, by identifying the Bible with the Word of God, Protestant orthodoxy attempted to divinize a human and worldly entity and to bind God’s freedom (Barth, 1954, p. 217). Church and the Bible become God’s Word when and as God freely chooses, but they do not thereby attain some permanently divine quality (Barth, 1977, p. 127). They remain in themselves wholly fallible, wholly human, and worldly (Barth, 1977, pp. 174–175).” This resolute criticism of religion extends back to the concept of marking a difference between the “visible” and the “invisible” church advocated for by St. Augustine (2000, Book I, Chapter 35) and re-embraced by the Protestant Reformation, notably by John Calvin (1960, Book IV, Chapter 1, pp. 1021–1022). The “visible” refers to people practicing their religion as seen from the outside, while the “invisible” refers to believers truly practicing their faith, which can only be seen from the inside and is known only to God.

The notion of the incomprehensibility of the transcendent appeared more recently in an even more extreme form with post-modern theologians’ concept of *deconstruction*, which introduces the radical idea that the person and the aim of any text become irrelevant once the text becomes subject to interpretation; here, the interpretation will be no more than a mere reflection of the interpreter’s personality, and there will be as many valid interpretations as there are interpreters. The determination of the “meaning” of a text becomes an arbitrary and relative act. Michel Foucault revealed the hidden oppressive nature of authoritative Bible commentary (Lynch, 2011), and Derrida (1973, 1989) drew attention to several contradictory biblical interpretations based on different ways of reading the same source. These thoughts reflect Barth’s emphasis on the wholly unreachable, incomprehensible, and uncontrollable nature of the transcendent from the other side of the problem: the human’s pluralist, relativist, ephemerous nature. This is the frame of thought in which dialectical theologians interpret the relationship between R/S and health.

From the dialectical theologians’ point of view, religion is measurable, but spirituality is not; the effect of religion on health is measurable and elicitable, but in itself is not relevant from a spiritual perspective because religion is just a human attempt, a human construct that is unable to reach the goal it was created for. In contrast, an authentic spiritual life remains under the authority of the Total Other and, therefore, remains irreproducible for humans. The significant negative outcomes reported by several R/S–health studies are painful reminders that religion can be a dangerous, noxious human endeavor. If the overall effect on health is still positive, it is because, as we know from God’s self-revelation in the Bible, it is His own *intention* to attract humankind to Him and endow them with a full healthy life. However, the ways in which He does so remain under his authority and, thus, cannot be reproduced or anticipated in any concrete encounter with the transcendent.

### Theology in Exile?

This article takes a fresh look at how findings on the relationship between religion, spirituality, and health can be interpreted through the lens of three different schools of theology. As shown above, these three schools represent phases on a spectrum in the degree to which they are cautious about religion in general, which explains the somewhat arbitrary decision of presenting only these three schools. It must be noted that these schools do not encompass the totality of the theological literature distilled over centuries of Christian tradition, and other theological schools may lend further nuance to the picture. However, this article aims to illustrate how theology can constructively participate in the interpretation of scientific results, paving the way for theology’s wider return to the discourse and its due seat at the table.

As deduced from the above, not all interventions intended to elicit a “spiritual” response fulfill the criteria defined as authentic spirituality. Several of them might be viewed as mere psychostimulants from a theologian’s perspective. Moreover, psychological interventions could have effects on health that are worth investigating from a scientific point of view, but not all may meet the definition of spirituality as defined by theology. On the other hand, some results that indicate harm to one’s health may be considered latent signs of real health in the way that theologians define this word.

Consequently, not only is the place of theology in the discussion of research in the natural sciences legitimized by the methodological tool it uses, it can also make meaningful contributions to ensure that the R/S–health relationship is better understood. Theologians can contribute to a more nuanced discussion in the interpretation of results; they can also help to implement better protocols in future studies focusing on the R/S–health relationship. Finally, they can help to achieve a better understanding of the clinical implications of basic research. For a brief outline of theologians’ recommendations for R/S–health research and implementation in clinical practice, see Table 1.

**Table 1:** Questions of theology for basic research on R/S–health and for spiritual interventions in clinical practice

Questions for basic research
Is the religious content participants are exposed to *authentic*?^a^ Have biblical passages been used?
Have efforts been made to *personalize* the religious message? Have there been attempts to measure the degree to which the message has been internalized?
Does the protocol leave enough room for *spontaneity*?
If the measured effect of a religious practice/spiritual intervention on health appears negative, has the possibility of *other interpretations* been checked? Have the participants’ own religious notions about health been evaluated?
Questions for clinical practice
Have the patient’s *assessment of their own current state of health *and *their wishes *been identified?
Has the patient’s *own religious and theological orientation* been evaluated?
Has it been *compared* to mainstream traditions of authentic spirituality?^b^ Have any discrepancies been identified?
Could the chaplain help the patient to *re-evaluate* their apparently definite condition? Could it be reframed as a hidden possibility, as part of a broader process of growth?

Studies/interventions that do not fulfill these criteria cannot be considered spiritual in the genuine theological sense of the word

^a^Sacred texts, symbols, chants, rites, etc., with Reformed Christian theologians’ explicit emphasis on using original biblical quotes

^b^If possible, by involving an expert in the field (i.e., the chaplain)

### Theoretical Implications

From their respective theological positions, scholars will have meaningful questions to address the natural sciences. Theologians who humbly view reality (as experienced with the senses) as being the will of their God (or who even consider the realm of nature as not just being created by God but also as serving His self-revelation) will also seek to confirm or falsify their biblically based theories in the answers scientists obtain from their empirical observations of the natural world.

The **evolutionist** will ask the scientist: What tools do the natural sciences have to evaluate whether the effect of a phenomenon in the natural world can be considered an accommodation serving only the immediate survival of the individual or the species, or whether it serves long-term goals for the sake of which the individual or species may even accept making a short-term sacrifice? What long-term goals can be identified in the process of evolution other than survival? In other words, what is the direction and what is the *unit* of evolution? Does the measured effect of a spiritual intervention on health have a tangible imprint of evolution, or is it just an ephemeral sign of an accommodation?

The **correlationist** will ask: Knowing the scope of wounds that interpersonal relationships can inflict on the body and soul, especially the early mother–child relationship, does the relationship between God and humans take place in another dimension? That is to say, will the impact of the God–human relationship on health overwrite the harmful effects, that is, the long-term imprints of wounds acquired through noxious human relations? Do the psycho-neuro-endocrine-immune correlates of spiritual interventions have the power to balance or overwrite the corresponding injuries acquired during early development? In other words, taking the theological theoretical frame seriously opens avenues for a series of scientific measurements investigating areas of the highest sociological significance; to cite an extreme example, investigating the question of whether faith and religion can compensate for early injuries even up to ensuring a healthy, full adult life for children who are separated from their parents at birth and brought up in foster homes.

The **dialectical** theologian will not ask anything from science, yet will always remind researchers to remain open to the unexpected and the unexplainable when studying spirituality because they examine the effects of a phenomenon that will never be in their full control to elicit. The idea that these theologians emphasize goes far beyond the framework of a theological school and evokes the most basic warning of several religious traditions. It is in this spirit that rabbi Samuel E. Karff warns: “[T]he Holy One does not submit to an experimental design” (Levin & Meador, Foreword, p. x).

### Methodological Implications: Implications for Basic Research Methods

To investigate the effect of spirituality on health, theologians will put forward the following practical questions to researchers:

The **evolutionist** will ask: Do researchers try to ensure that the measured/elicited spiritual effect serves the patient’s *growth*, or does it simply *stimulate* the patient? Is the religious content participants are exposed to *authentic*, or is religion merely used as a stimulant in the research setting?

The **correlationist** will ask: What efforts have been made to *personalize* the religious message? What does sociological, anthropological, and psychological research have to offer to help the full force of the transcendent penetrate the thick fabric of the ephemeral?

The **dialectical** theologian will ask: Do our protocols leave enough room for *spontaneity*, for life to manifest itself with all its mysteries, or do we lose the transcendent through the artificial effort of the mundane? Is a study about the effect of religion on health truly measuring the impact of the Total Other on a patient’s life, or is it losing its religious content in the genuine sense of the word while overloaded with all the constrained efforts to pull the person out of their limits by means of mere human efforts? In other words, coercion, even unintentionally or covertly applied in the framework of a scientific measurement of religion and health, is not only unethical (as shown below) but is also methodologically flawed because it suppresses the very phenomenon it wants to measure.

### Clinical Implications: Implications for Clinical Practice—Toward the “Theology of Possibility”

*Not* taking theological viewpoints into account in clinical practice also raises ethical issues. As stated above, not dealing with theology reflects an indirect resolution, even an involuntary standpoint from the physician’s perspective. Hence, negligence could be reflected, or the opposite: The implicit standpoint of liberal theology—which might or might not fully reflect the patient’s (albeit often implicit) standpoint or religious affiliation—is to blindly consider spiritual effects as elicitable at will and beneficial for the patient’s “health” as conceived of by the physician. This could lead us to cross the limits of coercion. In other words, unconsciously taking a spiritual–theological standpoint that is different from the patient’s choice raises medical-ethical issues, regardless of the physician’s spiritual convictions.

In contrast, studying theology in a nuanced way can help us understand the proper clinical implications of research outcomes for the R/S–health relationship. The different schools of theology help us to understand why R/S cannot be viewed as simply another factor that can be prescribed for patients to benefit their health.

The insights of the three major theological schools presented above can be harmonized in an overall view in that the closer we move from the whole population toward the individual, and from long-term to short-term effects, the more effects of R/S on health we can expect to be nuanced and unpredictable. This is because, while the goal of R/S is health in the religious sense of the word (including physical health), at times, renouncement and self-sacrifice are also part of one’s religious toolbox.

Unlike in the natural sciences, “long term” and “short term” in theology refer to lengths of time that are not specified since theology conceives of time by the measure of divine revelation, on the basis of which durations become relative to the eternity of God and the subjectivity of the human experience. For instance, the verb tense for the future and the present is the same in biblical Hebrew, which creates a feeling of certainty in God’s prophecies as if they have already been accomplished in the present, although they may take centuries to become truly realized. By contrast, a misunderstanding or a deadlock in one’s inner spiritual life (e.g., prolonged shame, anger, or grief) may last for years until it is suddenly resolved during a conversation with a psychotherapist in a private practice or with a chaplain at one’s bedside.

Assuming, based on ***evolutionist*** theologians’ perspective, that the main aspects with which to measure a person’s evolution are the degree of their proximity to God and the extent to which they are intimately related to the Divine, ***correlationist*** theologians point out that evolution toward the divine is a learning process. This process not only has possible setbacks characterizing all learning processes but also contains phases of sacrifice, including negative outcomes for health. Meanwhile, ***dialectical*** theologians stress that the ultimate personal experience of God is out of one’s control.

Thus, while all efforts should be made to minimize the negative side effects of R/S interventions, not all negative impacts should be considered side effects, and not all negative impacts should immediately be attributed to a bad application.

Lewis (2009) put it more simply, going so far as to state that even death might not, in all circumstances, be viewed as a failure but rather one milestone in the process of treatment: “Those who put themselves in His hands will become perfect, as He is perfect—perfect in love, wisdom, joy, beauty, health, and immortality. The change will not be completed in this life, for death is an important part of the treatment. How far the change will have gone before death in any particular Christian is uncertain” (p. 207).

Theology defines health as growth toward the divine with all temporary setbacks that such growth implies. Especially in the acute phases of a disease, when the effects of spiritual interventions on health become controversial, the theological viewpoint’s practical contribution to R/S interventions (regarding health) becomes embodied in the way that spiritual interventions *redefine* health. In particular, during the briefest spiritual interventions, which are represented in clinical practice by the chaplain’s interaction with the patient, in the theological sense, the major, decisive impact of spiritual interventions on health can be to redefine the very notion of health. Thus, the incomprehensible uncertainty of the “here and now” is reframed in the wider picture of belief in the evolution of the self toward the transcendent.

## Abbreviations

R/S: Religion/spirituality
JHWH: The name of God in the old testament (a sequence of consonants called the Tetragrammaton)

## References

[CR1] Augustine of Hippo (2000). The city of God (M. Dods, Trans.).

[CR2] Béres, A., Emri, M., Aranyi, C., Fajtai, D., Nagy, F., Szabó, P., Bödecs, P., Hörcsik, E., Perpékné Papp, É., Tomanek, F., Kuti, M., Petőfalviné, Á., Kisdeákné, H., Bíró, G., Kovács, D., Bakos, B., Vinczen, E., Gál, E., Sillinger, R., Szalai, Z., Szilágyi, A., Kiss-Merki, M., Nagyéri, G., Fodor, J., Németh, T., Papp, E., Repa, I. (2022). Healing through faith: Meeting a chaplain coupled with biblical readings could produce lymphocyte changes that correlate with brain activity (HEALING study). [version 4; peer review: 1 not approved]. *F1000Research, 10*, 1295. 10.12688/f1000research.74504.4 10.12688/f1000research.74504.5 PMC12149807 40496603

[CR3] Barth K (1933). The epistle to the Romans.

[CR4] Barth K (1954). Against the stream? Shorter post-war writings (1945–1952).

[CR5] Barth K (1977). Church dogmatics I.

[CR6] Barth K (1986). A Karl Barth reader.

[CR7] Bowker J, Bowker J (2000). Tillich, Paul Johannes Oskar (1886–1965). The concise Oxford dictionary of world religions.

[CR8] Bright J (2000). A history of Israel.

[CR9] Calvin J (1960). Institutes of the Christian religion (F. L. Battles, Trans.).

[CR10] Cobb JB (1962). Living options in protestant theology: A survey of methods.

[CR11] De Chardin PT (2008). The phenomenon of man (B. Wall, Trans.).

[CR12] Derrida J (1973). Speech and phenomena and other essays on Husserl’s theory of signs.

[CR13] Derrida J, Budick S, Iser W (1989). How to avoid speaking: Denials. Languages of the unsayable: The play of negativity in literature and literary theory.

[CR14] Groopman J (2004). God at the bedside. New England Journal of Medicine.

[CR15] Jung CG (1959). The archetypes and the collective unconscious.

[CR16] Koenig HG (2012). Religion, spirituality, and health: The research and clinical implications. ISRN Psychiatry.

[CR17] Levin J, Meador K (2012). Healing to all their flesh: Jewish and Christian perspectives on spirituality, theology, and health.

[CR18] Lewis CS (2009). Mere Christianity.

[CR19] Lynch RA, Taylor D (2011). Foucault’s theory of power. Michel Foucault: Key concepts.

[CR20] McGrath AE (1994). Christian theology: An introduction.

[CR21] Michaud D, Kujundzija Z, Cassell P (2005). Karl Barth (1886–1968).

[CR22] Otaiku AI (2022). Religiosity and risk of Parkinson’s disease in England and the USA. Journal of Religion and Health.

[CR23] Otto R (1923). The idea of the holy.

[CR24] Pannenberg, W. (2006). *Rendszeres teológia, II kötet* [*Systematic theology vol. 2*] (T. Görföl, Trans.). Osiris

[CR25] Pannenberg, W. (2009a). Revelation as history and as word of God. In G. W. Bromiley (Ed., Trans.), *Systematic theology* (Vol. 1, pp. 230–257). Eerdmans Publishing

[CR26] Pannenberg, W. (2009b). The Lord’s supper and Christian worship. In G. W. Bromiley (Ed., Trans.), *Systematic theology* (Vol. 3, pp. 283–336). Eerdmans Publishing.

[CR27] Polkinghorne J (2002). The God of hope and the end of the world.

[CR28] Russell CA (1973). Science and religious belief: A selection of recent historical studies.

[CR29] Salopek P (2013). To walk the world.

[CR30] Schjoedt U, Stødkilde-Jørgensen H, Geertz AW, Roepstorff A (2009). Highly religious participants recruit areas of social cognition in personal prayer. Social Cognitive and Affective Neuroscience.

[CR31] Schleiermacher F (1999). The Christian faith.

[CR32] Tillich P (1951). Systematic theology I.

[CR33] Tillich P (1957). Systematic theology II.

[CR34] Tillich P (1980). The courage to be.

[CR35] Tillich P (1981). The meaning of health.

[CR36] Tillich P, Church FF (1999). Invocation: The lost dimension in religion. The essential Tillich: An anthology of the writings of Paul Tillich.

[CR37] Unhjem, A. (2022). Paul Tillich. *Encyclopaedia Britannica*. https://www.britannica.com/biography/Paul-Tillich

[CR38] Weöres, S. (2012, April 13). Ten steps to entirety (D. R. Goldsmith, Trans.) [Status update]. Facebook. https://www.facebook.com/202672929755282/posts/386587751363798/

[CR39] Whitehead, A. N. (1978). *Process and reality*. The Free Press.

[CR40] Wikimedia Commons. (2023). File: The Scientific Method. svg. https://commons.wikimedia.org/wiki/File:The_Scientific_Method.svg#metadata

[CR41] Young JE, Klosko JS, Weishaar ME (2003). Schema therapy: A practitioner’s guide.

